# Functional Characterization of KNOX and BELL Genes in Temperature-Responsive Floral Morphogenesis of Passion Fruit (*Passiflora edulis*)

**DOI:** 10.3390/plants14101440

**Published:** 2025-05-12

**Authors:** Xinni Jiang, Jie Miao, Weifan Zu, Ruohan Zhou, Lexin Zheng, Ying Wei, Chunmei Lai, Rongjuan Qin, Ping Zheng, Xiuqing Wei, Jiahui Xu, Yuan Qin, Xiaoping Niu

**Affiliations:** 1 Fujian Provincial Key Laboratory of Haixia Applied Plant Systems Biology, College of Life Science, Fujian Agriculture and Forestry University, Fuzhou 350002, China; 2 Fishery Multiplication Management Station of Lijiang River Water Supply Hub Project, Guilin 541001, China; 3 Pingtan Science and Technology Research Institute, Fujian Agriculture and Forestry University, Pingtan 350400, China; 4 Fruit Research Institute, Fujian Academy of Agricultural Sciences, Fuzhou 350013, China

**Keywords:** *TALE* gene, evolutionary analysis, floral organogenesis, heat/cold stress

## Abstract

Passion fruit (*Passiflora edulis*), a tropical crop of significant economic value, exhibits temperature-sensitive floral development. Here, we identified 23 *TALE* transcription factors (*PeTALEs*) and characterized their roles in floral organogenesis and thermal adaptation. Phylogenetic analysis classified *PeTALEs* into *KNOX* and *BELL* subfamilies, with conserved domain architectures and cis-regulatory elements linked to stress and hormone signaling. Spatiotemporal expression profiling revealed *PeTALE21* as a key regulator of corona initiation, while *PeTALE17* dominated in later floral stages. Temperature stress assays demonstrated cold-induced upregulation of *PeTALE15*/*16*/*19*/*22* and heat-mediated suppression of *PeTALE10*/*18*/*21*. Yeast two-hybrid assays uncovered functional interactions between *PeTALE3*/*16*/*18*/*22*/*23*, highlighting a network governing floral thermoresilience. This study provides the first genome-wide analysis of *PeTALEs*, offering insights for breeding climate-resilient passion fruit varieties.

## 1. Introduction

Plants maintain their lifelong organogenic capacity through stem cell reservoirs localized in meristematic tissues [1]. The shoot apical meristem (SAM), serving as the primary source for leaf and axillary meristem formation, undergoes a developmental transition into the inflorescence meristem during flowering initiation, ultimately generating floral meristems (FMs) that develop into gynoecia, which are thought to be modified leaves, with their margins defining lateral organ boundaries [2]. This developmental continuity between SAM dynamics and fruit patterning highlights conserved regulatory mechanisms governing plant morphogenesis [2].

Meristematic cell proliferation and differentiation are precisely orchestrated by transcription factor networks, integrating positional cues, differentiation states, and growth signals [1,2]. Central to this regulation are KNOTTED1-like homeodomain (KNOX) proteins, first characterized in maize [3]. In *Arabidopsis*, the functional interplay between SHOOT MERISTEMLESS (STM) and WUSCHEL (WUS) governs stem cell maintenance [4]. WUS sustains the central stem cell niche, while STM preserves SAM undifferentiation by modulating hormone homeostasis [2]. STM belongs to the Three-Amino-acid-Loop-Extension (TALE) homeodomain superclass of transcription factors, which includes both KNOTTED-like (KNAT or KNOX) and BEL1-like (BLH or BELL) proteins [5]. These TALE factors typically function as KNOX–BELL heterodimers, regulating a wide array of developmental processes, including meristem maintenance, leaf development, and flower formation [6,7]. Specifically, STM maintains the undifferentiated state of meristematic cells by repressing gibberellin (GA) biosynthesis, promoting GA catabolism, and stimulating cytokinin (CK) biosynthesis [8,9,10]. Furthermore, *STM* negatively regulates ASYMMETRIC LEAVES1 (*AS1*), a MYB transcription factor that antagonizes other TALE members (*KNAT1*/*BREVIPEDICELLUS*, *KNAT2*, and *KNAT6*) to regulate leaf patterning [2,11]. Lateral organ initiation requires the coordinated regulation of auxin and GA signaling alongside the downregulation of STM-related factors [7].

The TALE family, comprising KNOX and BELL subfamilies, features conserved structural domains: bipartite KNOX/BELL interaction domains upstream of the homeodomain, with KNOX proteins containing an ELK domain and BELL proteins possessing SKY and ZIBEL domains [6]. These proteins form functional heterodimers through KNOX–BELL domain interactions, regulating processes ranging from meristem maintenance to stress adaptation [12,13]. KNOX members (e.g., STM) predominantly govern meristem activity and organ patterning, whereas BELL proteins specialize in floral development and organ differentiation [12,13]. Mechanistically, TALE factors coordinate hormone signaling pathways, tuber formation, and stress response [14,15,16,17,18,19]. In *Arabidopsis*, *KNOX* genes are predominantly expressed in meristematic regions, regulating key processes such as cytokinin biosynthesis essential for maintaining SAM activity [10]. KNOX proteins are also implicated in secondary cell wall biosynthesis in poplar [20]. Additionally, *KNOX* and *BELL* genes play significant roles in regulating hormone pathways, such as those for gibberellins (GAs), abscisic acid (ABA), and cytokinin (CK), which are vital for plant growth and stress responses [21]. For example, the AtBLH1 and AtKNAT3 proteins form heterodimers to regulate seed germination through ABA signaling [22]. *KNOX* genes in *A. thaliana* influence CK biosynthetic gene expression to maintain SAM development [10], while in apple (*Malus domestica*), *KNOX* genes activate the ABA signaling pathway, promoting callus formation [23]. In response to abiotic stress, TALE gene expression is modulated by environmental stimuli. For example, soybean TALE gene promoters contain *cis*-elements responsive to salt and drought stresses [24]. Similarly, the overexpression of the KNOX-like gene *TaKNOX11a* from *Triticum aestivum* in *A. thaliana* enhances stress tolerance by modulating proline content and reducing oxidative damage [24]. These findings highlight that *TALE* genes regulate not only developmental processes but also plant adaption to environmental stresses.

Floral initiation, a critical step in the transition from vegetative to reproductive growth, is governed by a complex network of genetic regulators that integrate endogenous and environmental cues [25,26]. Temperature, in particular, plays a key role in floral induction and organ differentiation [27,28]. Plants must accurately interpret temperature signals over various timescales to synchronize development with seasonal changes. Although the mechanisms underlying acute thermal stress responses are relatively well-studied [29,30], the processes by which plants integrate long-term temperature signals to regulate floral development remain less understood. Passion fruit (*Passiflora edulis*), a tropical crop valued for its nutritional and economic significance [31], offers a unique system for studying temperature-sensitive floral organogenesis. Introduced to southern China in 1901, it achieves yields of up to 15 tons per hectare annually [32], yet exhibits notable thermal sensitivity: flower bud abortion at high temperatures (30/25 °C) and suppressed bud formation at cooler temperatures (20/15 °C) [33,34,35]. Its complex floral architecture (sepals, petals, corona, stamens, and carpels) makes it an excellent model for developmental studies.

Despite these attributes, the role of the floral TALE gene family in the thermal regulation of floral development in *P. edulis* remains unexplored. In this study, we conducted the first genome-wide analysis of *PeTALE* genes, combining RNA-seq profiling of temperature-stressed buds with structural, phylogenetic, and promoter analyses. Given their established roles in meristem identity, floral organogenesis, and hormone signaling, we focused on the KNOX and BELL subfamilies for functional analysis. We systematically investigated their chromosomal distribution, predicted protein interaction networks, and associations with hormone-responsive pathways to elucidate their functions in temperature-modulated floral development. This work provides a molecular framework for understanding thermal adaptation mechanisms in passion fruit and offers critical insights for breeding climate-resilient varieties.

## 2. Results

### 2.1. Identification of the TALE Gene Family in Passion Fruit

To identify candidate TALE members in *P. edulis*, we conducted a comprehensive search using the HMMER profile of TALE proteins as queries against the passion fruit protein database via the BLASTP algorithm. The resulting *PeTALE* candidate genes were further validated using the Pfam and SMART databases to confirm the presence of the characteristically conserved domains, including the homeodomain (HD) and KNOX or BEL1-like domains. Ultimately, a total of 23 *PeTALE* genes were identified and designated *PeTALE1* to *PeTALE23* based on their chromosomal positions (Table 1). Notably, the majority of these genes (10 out of 23) were located on chromosome LG01, while only a single gene was found on chromosome LG05, suggesting potential clustering of TALE family members on specific chromosomes (Figure S1). This clustering pattern may indicate gene duplication events or the existence of conserved genomic regions harboring multiple TALE genes, which could be linked to the regulation of key developmental processes. For a detailed analysis of the physical and biochemical properties of the PeTALE proteins, several key parameters were calculated, including protein length, molecular weight (MW), isoelectric point (pI), aliphatic index (A.I.), and predicted subcellular localization (Table 1). PeTALE protein lengths exhibited considerable variation, ranging from 195 amino acids (PeTALE11) to 811 amino acids (PeTALE21), reflecting functional diversity within the family. Correspondingly, molecular weight ranged from 22.506 kDa (PeTALE11) to 89.581 kDa (PeTALE21). The predicted isoelectric points varied from 4.70 (PeTALE5) to 8.75 (PeTALE22), indicating variability in charge properties across family members. We also assessed protein stability and hydrophobicity. According to the instability index [36], PeTALE1 was classified as a stable protein (instability index < 40), whereas the remaining members were predicted to be unstable. The aliphatic index (A.I.), which correlates with thermal stability, ranged from 60.12 (PeTALE6) to 83.99 (PeTALE15), suggesting that some PeTALE proteins may exhibit greater thermal stability than others. Furthermore, the grand average of hydropathicity (GRAVY) values, which ranged from −0.923 (PeTALE11) to −0.385 (PeTALE19), indicated that all identified PeTALE proteins are hydrophilic, potentially influencing their interaction with other hydrophilic molecules or their nuclear localization. Subcellular localization predictions revealed that all PeTALE proteins are likely localized to the nucleus (Table 1), consistent with their putative roles as transcription factors regulating gene expression during critical stages of plant development. 

### 2.2. Classification and Phylogenetic Relationships of PeTALEs

**Figure 1:**
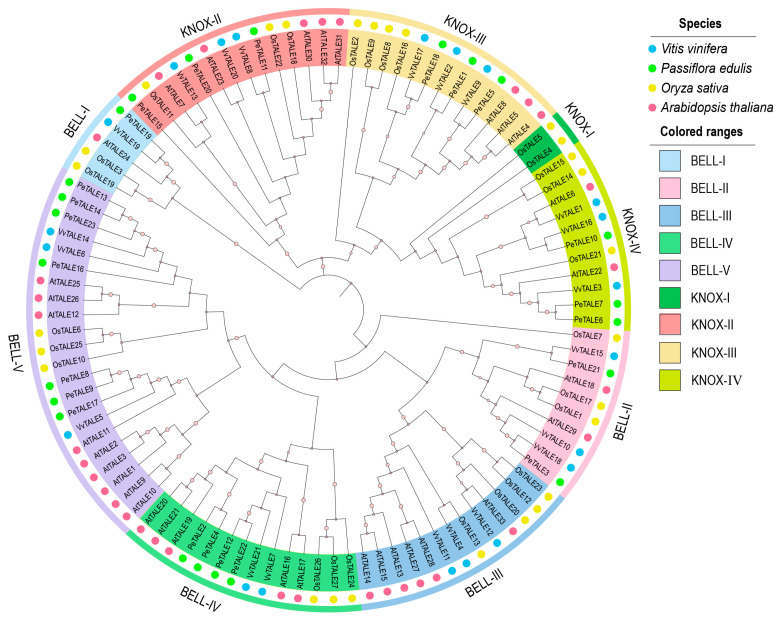
Phylogenetic relationships of the *TALE* gene family across *Passiflora edulis* and other plant species. The unrooted phylogenetic tree was constructed using the *TALE* gene sequences from *Passiflora edulis* (green dots), *Vitis vinifera* (blue dots), *Oryza sativa* (yellow dots), and *Arabidopsis thaliana* (red dots), employing the neighbor-joining (NJ) method. The tree highlights well-supported clades, with bootstrap values greater than 95% indicated by yellow cycles. Based on phylogenetic clustering and bootstrap support, the *TALE* genes are classified into two major groups: KNOX and BELL.

To explore the evolutionary relationships and classification of TALE proteins in *P. edulis*, we constructed a phylogenetic tree using the neighbor-joining (NJ) method. The analysis included 104 TALE proteins from three species—*Vitis vinifera* (21 proteins), *Arabidopsis thaliana* (33 proteins), and *Oryza sativa* (27 proteins)—along with the 23 PeTALE proteins identified in this study. The resulting tree revealed two major clades, corresponding to the KNOX and BELL subfamilies, which were further subdivided into KNOX-I to KNOX-IV and BELL-I to BELL-V, respectively, based on specific domain signatures. Among the 23 *PeTALE* genes, 14 were assigned to the BELL subfamily, characterized by the presence of both a POX domain and a homeodomain, while the remaining 9 *PeTALE* genes were categorized within the KNOX subfamily, defined by the presence of the KNOX1 or KNOX2 domains, an ELK motif, and a homeodomain. Within the BELL subfamily, the largest cluster was BELL-V, containing more than 20 TALE genes, whereas BELL-I had the fewest members. The intermediate groups, BELL-II, BELL-III, and BELL-IV, each harbored a moderate number of genes. In the KNOX subfamily, we observed a relatively even distribution across the subgroups: KNOX-I contained only two TALE members, while KNOX-II, KNOX-III, and KNOX-IV each included a larger number of TALE genes (Figure 1). The distribution of *PeTALE* genes across these subfamilies and subgroups reflects the evolutionary diversification of this gene family in *P. edulis*. Notably, the BELL subfamily, typically associated with floral development and organ identity regulation, appears to have undergone greater expansion in passion fruit compared to the KNOX subfamily, which is mainly linked to shoot and leaf development.

To assess the evolutionary pressure acting on the *PeTALE* genes, we calculated the non-synonymous (*Ka*) to synonymous (*Ks*) substitution ratio (*Ka*/*Ks*). This ratio provides insights into the selective forces shaping gene evolution: a *Ka*/*Ks* ratio less than 1 indicates purifying selection, a ratio equal to 1 suggests neutral selection, and a ratio greater than 1 is indicative of positive selection. Our analysis revealed that all *PeTALE* genes exhibited *Ka*/*Ks* ratios of less than 1 (Table 2), suggesting that these genes have predominantly undergone purifying selection throughout their evolutionary history.

### 2.3. Chromosomal Distribution and Synteny Analysis of PeTALE Genes

The chromosomal distribution of the 23 identified *PeTALE* genes was mapped based on the *P. edulis* genome sequences. The results revealed that the *PeTALE* genes are dispersed across multiple chromosomes, with each chromosome harboring one or more genes. Notably, chromosome 1 (LG01) contains the highest number of *PeTALE* genes, hosting 10 out of 23 (43.48%), while chromosome 5 (LG05) is home to only a single gene (4.35%). Chromosome 2 (LG02) contains 4 genes (17.39%), and chromosomes LG06, LG07, and LG08 each harbor 2 to 3 *PeTALE* genes. Interestingly, no *PeTALE* genes were located on chromosomes LG03, LG04, and LG09 (Figure S1), suggesting that gene loss or chromosomal rearrangements may have occurred during the evolution of the *TALE* gene family in *P. edulis*.

**Figure 2:**
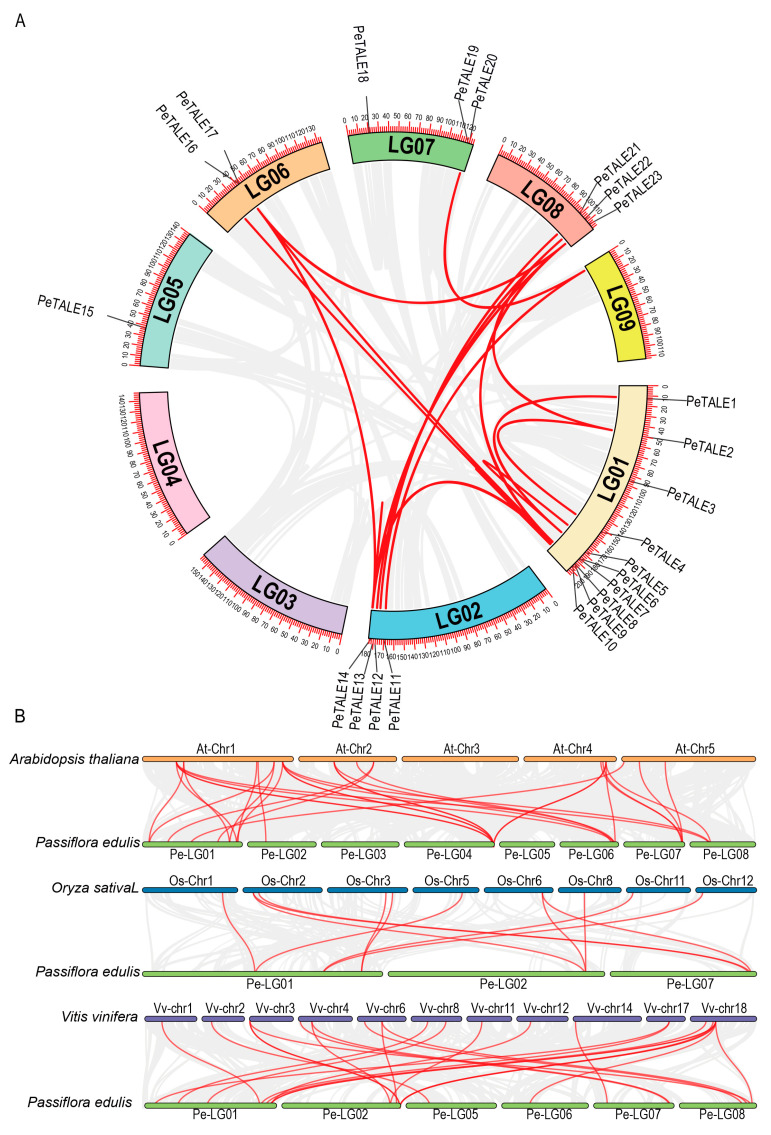
Intra- and inter-species collinearity analysis of *PeTALE* genes in *P. edulis*. (**A**) Gene duplication events in *P. edulis*: the syntenic blocks within the *P. edulis* genome are represented by gray background lines, with segmental or tandem duplication events of *PeTALE* genes highlighted by red lines. (**B**) Collinearity analysis across plant species: The diagram illustrates the collinearity relationships between TALE genes from *Arabidopsis thaliana*, *Oryza sativa*, *Vitis vinifera*, and *P. edulis*. Red lines connect orthologous *PeTALE* genes across these species, while gray lines indicate the broader collinear blocks shared among these species’ genomes.

To explore the mechanisms driving the expansion of the *PeTALE* gene family, we conducted a synteny analysis to identify both intra- and inter-species duplications. Intra-species synteny analysis revealed 18 pairs of duplicated *PeTALE* genes, indicative of gene duplication events within the *P. edulis* genome. Notable gene pairs include *PeTALE8*/*PeTALE9*, *PeTALE2*/*PeTALE4*, *PeTALE1*/*PeTALE5*, *PeTALE8*/*PeTALE13*, *PeTALE8*/*PeTALE14*, *PeTALE9*/*PeTALE13*, *PeTALE9*/*PeTALE14*, *PeTALE9*/*PeTALE16*, *PeTALE8*/*PeTALE17*, *PeTALE8*/*PeTALE23*, *PeTALE9*/*PeTALE23*, *PeTALE2*/*PeTALE22*, *PeTALE13*/*PeTALE14*, *PeTALE13*/*PeTALE16*, *PeTALE13*/*PeTALE23*, *PeTALE14*/*PeTALE23*, *PeTALE12*/*PeTALE22*, and *PeTALE16*/*PeTALE23* (Figure 2A; Table S1). Tandem duplicates were also evident, particularly in the *PeTALE6*/*PeTALE7* and *PeTALE16*/*PeTALE17* clusters, highlighting the role of local duplications in the expansion of this gene family (Figure S1). Inter-species synteny comparisons further revealed significant evolutionary relationships between *P. edulis* and other species. In particular, 26 syntenic gene pairs were identified between *P. edulis* and *Vitis vinifera*, such as *PeTALE5/VvTALE9*, *PeTALE10/VvTALE16*, *PeTALE5/VvTALE2*, *PeTALE1/VvTALE2*, *PeTALE8/VvTALE5*, *PeTALE9/VvTALE5*, *PeTALE6/VvTALE3*, *PeTALE3/VvTALE18*, *PeTALE2/VvTALE7*, *PeTALE11/VvTALE8*, *PeTALE13/VvTALE5*, *PeTALE14/VvTALE5*, *PeTALE13/VvTALE4*, *PeTALE14/VvTALE14*, *PeTALE13/VvTALE14*, *PeTALE11/VvTALE20*, *PeTALE12/VvTALE21*, *PeTALE15/VvTALE13*, *PeTALE16/VvTALE5*, *PeTALE18/VvTALE17*, *PeTALE19/VvTALE19*, *PeTALE20/VvTALE20*, *PeTALE23/VvTALE5*, *PeTALE23/VvTALE14*, *PeTALE21/VvTALE15*, and *PeTALE22/VvTALE21* (Figure 2B; Table S2). Among these, *PeTALE13* exhibited synteny with three distinct *VvTALE* genes (*VvTALE5*, *VvTALE4*, and *VvTALE14*), suggesting a complex pattern of gene conservation and divergence. A similar synteny analysis with *Arabidopsis thaliana* identified 32 gene pairs, including *PeTALE8/AtTALE11*, *PeTALE5/AtTALE8*, *PeTALE1/AtTALE5*, *PeTALE5/AtTALE5*, *PeTALE10/AtTALE6*, *PeTALE1/AtTALE8*, *PeTALE9/AtTALE3*, *PeTALE2/AtTALE20*, *PeTALE4/AtTALE20*, *PeTALE3/AtTALE29*, *PeTALE15/AtTALE7*, *PeTALE14/AtTALE10*, *PeTALE14/AtTALE3*, *PeTALE13/AtTALE10*, *PeTALE13/AtTALE3*, *PeTALE14/AtTALE12*, *PeTALE13/AtTALE12*, *PeTALE14/AtTALE10*, *PeTALE13/AtTALE26*, *PeTALE14/AtTALE26*, *PeTALE23/AtTALE10*, *PeTALE23/AtTALE3*, *PeTALE23/AtTALE12*, *PeTALE22/AtTALE17*, *PeTALE23/AtTALE26*, *PeTALE20/AtTALE23*, *PeTALE19/AtTALE24*, *PeTALE20/AtTALE30*, *PeTALE20/AtTALE31*, *PeTALE16/AtTALE10*, *PeTALE16/AtTALE3*, and *PeTALE16/AtTALE26* (Figure 2B; Table S2). These results underscore the evolutionary relationships between *PeTALE* genes in passion fruit and those in distantly related species like grapevine and *Arabidopsis*, pointing to shared ancestral origins and functional conservation across these plant species. These synteny analyses not only highlight the presence of both tandem and segmental duplications within the *PeTALE* gene family but also provide insights into the evolutionary processes shaping the expansion and diversification of *TALE* genes in *P. edulis*. The conservation of *PeTALE* gene pairs across species further suggests that these genes have maintained crucial developmental functions over evolutionary time.

### 2.4. Conserved Motifs and Gene Structure Characteristic Analysis of PeTALE Genes

**Figure 3:**
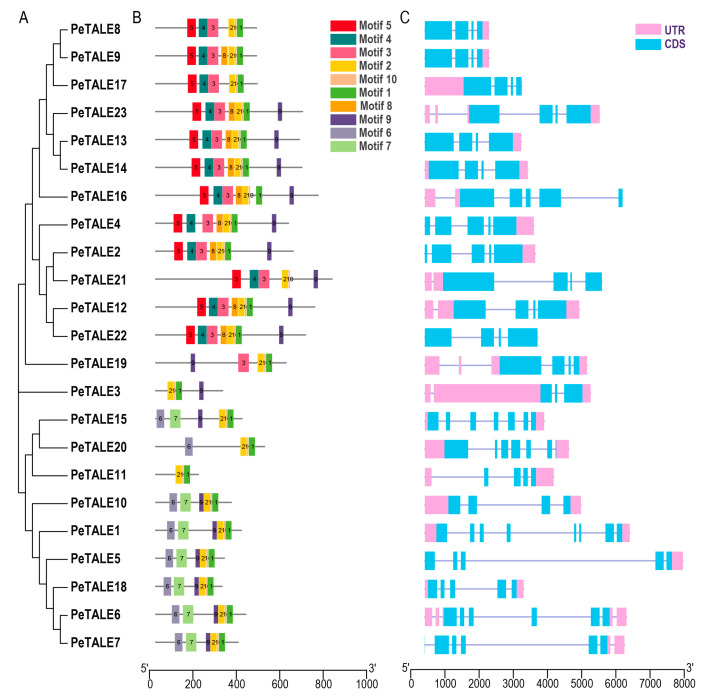
Gene structures and motif analysis of *PeTALE* genes in *P. edulis*. (**A**) A neighbor-joining (NJ) tree depicting the phylogenetic relationships among 23 TALE genes in *P. edulis*. (**B**) The conserved protein motifs within *PeTALE* genes are represented by color-coded boxes, each corresponding to a distinct motif. (**C**) Gene structures of *PeTALE* genes; the CDS regions are indicated in blue, the 5′ and 3′-UTR regions are shown in pink, and the introns are marked by purple lines.

The structural characteristics of *PeTALE* genes were examined by analyzing the conserved motifs and intron–exon organization of their coding sequences using the GFF annotation file from the *P. edulis* genome (Figure 3). A total of ten conserved motifs were identified across the *PeTALE* family. The core TALE domain, essential for the functional properties of most PeTALE proteins, was characterized by the presence of Motifs 1, 2, 9, and 10 (Figure 3B). However, certain motifs were either absent or uniquely present in specific PeTALE proteins. For example, PeTALE11 was composed solely of Motifs 1, 2, and 10, while Motif 9 was missing in PeTALE8, PeTALE9, PeTALE11, PeTALE17, and PeTALE20. These variations in motif composition suggest that different PeTALE proteins may fulfill distinct biological functions, potentially modulated by specific motifs in response to particular cellular or environmental cues.

In addition to motif conservation, our analysis revealed that genes within the same subfamily often share similar structure features. For instance, five *PeTALE* genes (*PeTALE8*, *PeTALE9*, *PeTALE13*, *PeTALE14*, and *PeTALE17*) each harbor three introns, whereas *PeTALE2* and *PeTALE21* possess four introns, and *PeTALE15* and *PeTALE20* are characterized by six introns (Figure 3C). Furthermore, 12 *PeTALE* genes (*PeTALE1*, *PeTALE3*, *PeTALE6*, *PeTALE10*, *PeTALE11*, *PeTALE12*, *PeTALE14*, *PeTALE15*, *PeTALE18*, *PeTALE19*, *PeTALE20*, and *PeTALE23*) contain both 5′ and 3′-UTRs, while 8 genes (*PeTALE2*, *PeTALE4*, *PeTALE5*, *PeTALE7*, *PeTALE8*, *PeTALE9*, *PeTALE13*, and *PeTALE22*) lacked a 5′-UTR, and 3 genes (*PeTALE16*, *PeTALE17*, and *PeTALE21*) lacked a 3′-UTR (Figure 3C). Notably, gene structure tended to be highly conserved within the same clade, while greater variation was observed between clades. For instance, genes such as *PeTALE8*, *PeTALE9*, *PeTALE13*, *PeTALE14*, and *PeTALE17*, which share a common intron–exon structure pattern (three introns), were clustered together (Figure 3C), suggesting a potential link between gene structure and evolutionary lineage.

**Figure 4:**
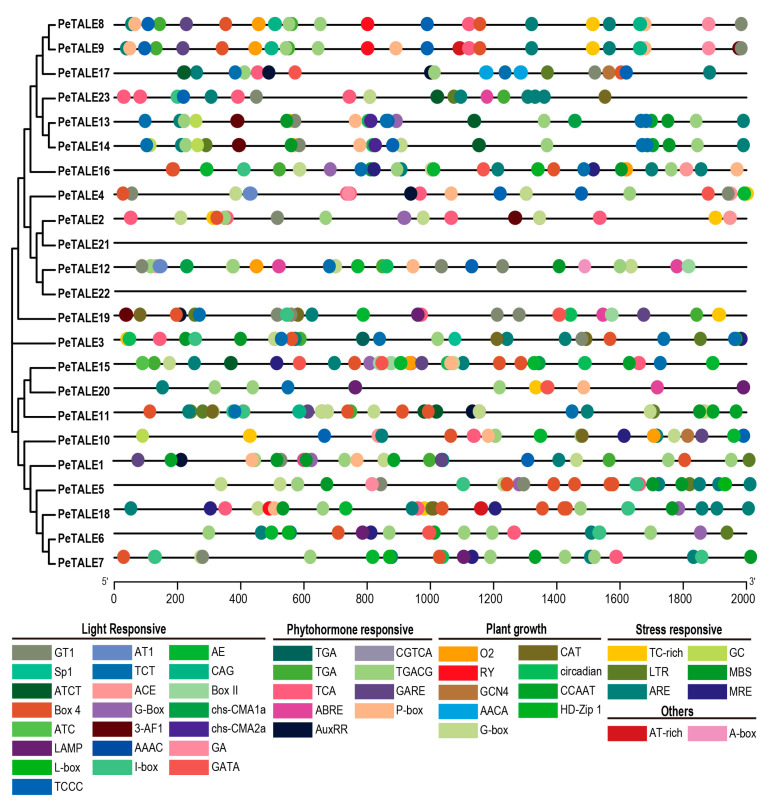
Cis-element analysis of *PeTALE* gene promoters in *P. edulis*. A total of five distinct types of cis-elements were detected in the 2000 bp upstream promoters of *PeTALE* genes, each associated with various biological processes, including light response, phytohormone regulation, stress response, and growth.

To further investigate the potential regulatory mechanisms of *PeTALE* genes, we examined the cis-elements within their promoter regions, defined as the 2000 bp sequence upstream of the transcriptional start codon. This analysis revealed a diverse array of cis-elements, including those associated with plant growth (9 elements), stress responses (6 elements), light signaling (22 elements), as well as unique elements such as an AT-rich DNA-binding protein (ATBP-1) binding site and an A-box element (Figure 4). Most *PeTALE* genes (except *PeTALE21* and *PeTALE22*) contained at least one light-responsive cis-element in their promoter regions, indicating a potential role in light-mediated gene regulation. Additionally, the majority of *PeTALE* genes (with the exceptions of *PeTALE21*, *PeTALE15*, *PeTALE11*, *PeTALE23*, and *PeTALE22*) contained an MeJA-responsive cis-acting element, suggesting that these genes may participate in phytohormone signaling pathways, particularly in response to MeJA, which is crucial for plant defense mechanisms and stress responses.

### 2.5. The Secondary Structure Prediction of PeTALE Proteins

**Figure 5:**
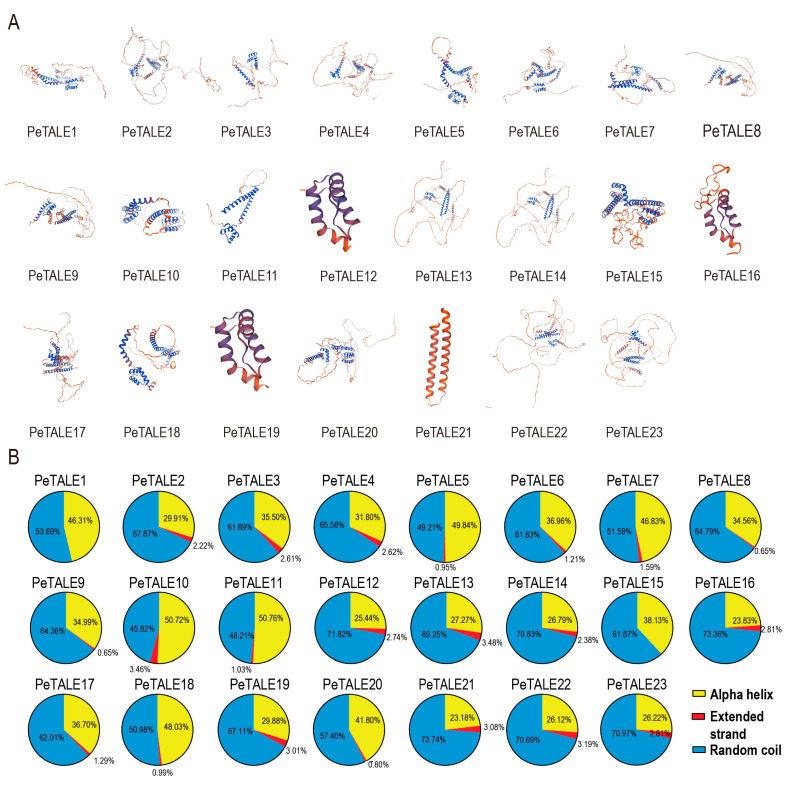
Predicted 3D structural models and secondary structure composition of PeTALE proteins. (**A**) 3D structural models of PeTALE proteins generated based on secondary structure predication. (**B**) Distribution of secondary structural elements (alpha helix, extended strand, and random coil) of each PeTALE protein.

Understanding protein structure is fundamental for elucidating its functional roles and cellular localization. Three-dimension (3D) structural models of PeTALE proteins from different subfamilies were generated using the SWISS-MODEL platform, and the quality of each model was evaluated based on the GMQE and Q-mean scores. These quality metrics were used to select the most reliable models for further analysis. The results revealed that, while there was notable variability in the 3D structures of PeTALE proteins across subfamilies, proteins within the same subfamily displayed highly conserved spatial configurations (Figure 5A). For instance, PeTALE8 and PeTALE9, PeTALE2 and PeTALE19, and PeTALE22 and PeTALE23 exhibited similar secondary structure arrangements, supporting the hypothesis that proteins within closely related groups share common structural features (Figure 5A).

The structural diversity among these proteins is strongly influenced by their secondary structure composition. To explore this further, we conducted a secondary structure prediction analysis for the PeTALE proteins. The results revealed that the majority of PeTALE proteins are predominantly composed of alpha helices, extended strands, and random coils, with the respective proportions ranging from 23.18% to 50.77%, 0.65% to 3.48%, and 45.82% to 73.74%, respectively (Figure 5B). Interestingly, members of the KNOX subfamily, such as PeTALE1 and PeTALE15, exhibited distinct structural profiles characterized by a complete absence of extended strands and a substantial increase in random coil content, averaging 62.39%. In contrast, the alpha helix structure remained the most prevalent overall, accounting for an average of 35.72% of the total sequence, while extended strands represented only about 2.07% (Figure 5B).

### 2.6. Expression Profiling of PeTALE Genes For Different Floral Organs and Their Response to Temperature

**Figure 6:**
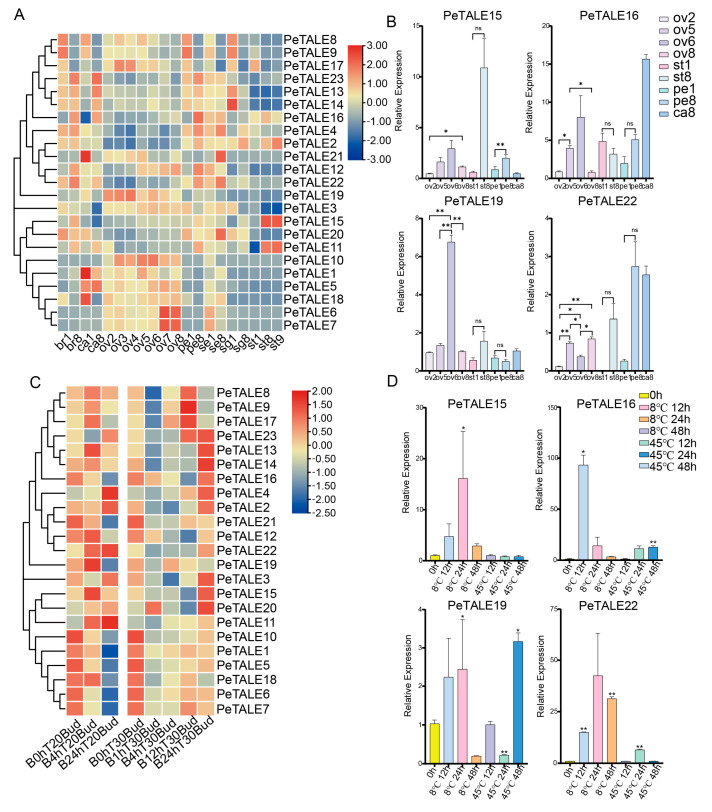
Expression profiles of *PeTALE* genes during floral organ formation and temperature stress response. (**A**,**B**) Heatmaps and qRT-PCR displaying the expression profiles of 23 *PeTALE* genes across 7 floral organs of *P. edulis*: bract (Br), corona (Ca), ovules (Ov S2-S8), petals (Pe), sepals (Se), stigmas (Sg), and stamens (St). (**C**) Expression of *PeTALE* genes in floral buds subjected to low-temperature (20 °C) and high-temperature (30 °C) conditions, as determined through RNA-seq analysis. (**D**) Quantitative RT-PCR validation of selected *PeTALE* gene expression levels under cold and heat stress. The control group consists of buds maintained at normal temperature conditions. Relative gene expression was quantified using the 2^−∆∆Ct^ method, with statistical significance indicated by * (*p* ≤ 0.05) and ** (*p* ≤ 0.01), as determined by Student’s *t*-test. Error bars represent the mean ± SD.

To gain insight into the potential roles of the 23 *PeTALE* genes, their expression patterns were analyzed using RNA-seq data from various tissues and developmental stages in *P. edulis.* Expression levels were assessed across a range of tissues, including bracts (br1 and br8), coronas (Ca1 and Ca8), ovules (Ov S2-S8), petals (Pe1 and Pe8), sepals (Se1 and Se8), stigmas (Sg1 and Sg8), and stamens (St1, St8, and St9). Distinct expression profiles were observed across the different *PeTALE* subfamilies. Genes from the BELL subfamily exhibited diverse expression patterns. For instance, *PeTALE8*, *PeTALE9*, *PeTALE13*, *and PeTALE14* (BELL-II group) were highly expressed during the early developmental stages, whereas *PeTALE2* and *PeTALE4* (also BELL-II group) showed enhanced expression during the mid- and late stages of stigma development (Figure 6A), suggesting their potential involvement in stigma morphogenesis. Several BELL genes, such as *PeTALE13* and *PeTALE14*, were strongly expressed during early stigma development, as well as in petals and during the final stages of corona development. Similarly, *PeTALE8* and *PeTALE9* exhibited elevated expression during early stages of petal, corona, and stigma development and throughout most stages of ovule development. Notably, *PeTALE21* showed strong, specific expression during the early stages of corona development. Within the BELL subfamily, genes such as *PeTALE23*, *PeTALE16*, *PeTALE4*, and *PeTAL22* displayed preferential expression in sepals, with slightly higher levels also observed in petals compared to other tissues. *PeTALE17* was notably expressed at high levels during early petal, stigma, stamen, and ovule development (Figure 6B). In contrast, *PeTALE4* showed strong expression during the late stages of corona and stamen development, with moderate expression throughout the petal and sepal stages. Although *PeTALE22* exhibited relatively lower expression in stamens, stigmas, and ovules, it demonstrated higher expression during certain stages of corona development, aligning with the expression profile of *PeTALE4*. The expression of some *PeTALE* genes was highly tissue-specific. For instance, *PeTALE19* and *PeTALE3* were predominantly expressed across ovule development stages, while *PeTALE11* and *PeTALE15* were highly expressed only during later stages of stamen development. Moreover, the expression levels of several genes exhibited temporal variation. For example, *PeTALE13*, *PeTALE14*, and *PeTALE16* showed progressively increasing expression during corona and petal development, whereas *PeTALE22* exhibited a similar upward trend specifically in sepals (Figure 6B).

To assess the temperature sensitivity of *PeTALE* genes, RNA-seq data were analyzed from flower buds exposed to cold (20 °C) and heat (30 °C) stress across different time points (Figure 6C). After excluding genes with low expression, notable differences emerged under temperature stress. Cold stress led to the upregulation of genes such as *PeTALE11*, *PeTALE20*, *PeTALE3*, *PeTALE22*, *PeTALE2*, *PeTALE23*, and *PeTALE4*, with *PeTALE11*, *PeTALE3*, *PeTALE4*, and *PeTALE23* exhibiting significant increases as treatment duration increased (Figure 6C). Conversely, heat stress responses were more nuanced. While most genes initially showed elevated expression, some (*PeTALE8*, *PeTALE9*, *PeTALE17*, and *PeTALE19*) were upregulated at the 1 and 4 h time points but downregulated after 12 and 24 h. Additionally, genes such as *PeTALE23*, *PeTALE13*, *PeTALE4*, *PeTALE22*, *PeTALE15*, and *PeTALE20* peaked at specific times, while *PeTALE10*, *PeTALE18*, and *PeTALE21* were repressed under heat conditions (Figure 6D). These findings suggest that specific *PeTALE* subfamilies exhibit distinct temperature-responsive behaviors: BELL-II genes (e.g., *PeTALE3*) and BELL-IV genes (e.g., *PeTALE4*) primarily respond to cold stress, whereas KNOX-IV (*PeTALE10*), KNOX-III (*PeTALE18*), and certain BELL-II genes (e.g., *PeTALE21*) are more responsive to heat stress. Together, these results offer valuable insights into the roles of *PeTALE* genes in developmental regulation and stress responses in *P. edulis*, providing a foundation for future functional studies on plant growth and adaptation.

### 2.7. Protein Interaction of PeTALE Proteins

**Figure 7:**
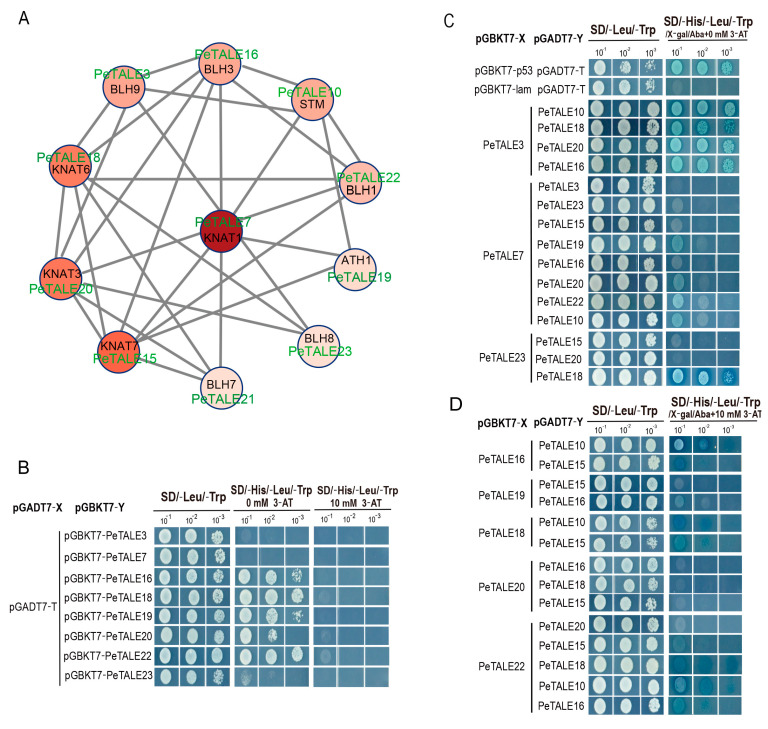
Predicted and verified functional interaction networks of PeTALE proteins. (**A**) Predicted functional interactions of PeTALE proteins, based on orthologous relationships with Arabidopsis genes. The eleven identified interactions are depicted, with orthologs marked in green. The strength of correlations between node genes is represented by color gradients, indicating varying levels of interaction strength. Light gray lines represent the interaction links between PeTALE proteins and other associated proteins, highlighting potential regulatory or structural interactions within the network. (**B***–***D**) Confirmation of these protein–protein interactions through yeast two-hybrid (Y2H) assays.

To further investigate the functional conservation of the *PeTALE* gene family, we performed a protein–protein interaction (PPI) analysis to predict potential interacting partners of PeTALE proteins (Figure 7A). The analysis revealed that *PeTALE7*, an ortholog of *AtKNAT1*, exhibited high expression in ovules and formed a robust interaction network with multiple PeTALE proteins, based on their *Arabidopsis* orthologs (including *STM*, *ATH1*, *BLH1/3/7/8/9*, and *KNAT3/6/7*). This interaction cluster (comprising *PeTALE7/8/9/10/15/16/18/19/20/21/22/23*) suggests a conserved role for *PeTALE7* and its associated genes in floral organogenesis and reproductive tissue development (Figure 7A). Additionally, we identified 10 orthologous proteins (*STM*, *ATH1*, *BLH1/3/7/8/9*, and *KNAT3/6/7*) as candidate interactors of *PeTALE3/7/16/18/19/20/22/23*, with varying interaction strengths observed among them (Figure 7A).

To validate these interactions, yeast two-hybrid (Y2H) assays were conducted. Initial transcriptional activity screening revealed that all transformed yeast strains grew normally on SD/-Leu-Trp medium (Figure 7B). Five co-transformed yeast strains (*pGBKT7-PeTALE16/18/19/20/22*) exhibited growth on SD/-His-Leu-Trp medium (0 mM 3-AT) and displayed blue coloration on X-α-Gal-supplemented SD/-His-Leu-Trp medium (0 mM 3-AT), confirming their transcriptional activation capacity. However, growth was suppressed on SD/-His-Leu-Trp medium supplemented with 10 mM 3-AT, indicating transcriptional inhibition (Figure 7B). In contrast, three plasmids (*pGBKT7-PeTALE3/7/23*) showed no growth or coloration under the same conditions, confirming their lack of transcriptional activation. Subsequent Y2H assays for 29 potential KNOX–BELL combinations demonstrated direct interactions between PeTALE3 and three KNOX proteins (PeTALE10/18/20), as well as one BELL protein (PeTALE16), while PeTALE18 interacted with two BELL proteins (PeTALE23 and PeTALE22) (Figure 7C,D). Weak interactions were observed between PeTALE7 and PeTALE19/20/22/10, as well as among PeTALE10–PeTALE16, PeTALE10–PeTALE18, and PeTALE10–PeTALE22 combinations (Figure 7C,D). These findings highlight a complex interaction network among PeTALE proteins, emphasizing their potential regulatory roles in floral organogenesis and reproductive tissue development.

## 3. Discussion

### 3.1. Evolutionary Conservation and Diversification of PeTALE Genes

As a globally significant horticultural crop, *Passiflora edulis* offers unique advantages for studying floral organogenesis due to its intricate flower morphology and temperature-sensitive reproductive development [34]. The *TALE* gene family is widely distributed across plant genomes and plays a crucial role in regulating various aspects of plant growth, development, and stress responses. Previous genome-wide analyses of *TALE* genes have been conducted in numerous species, including *Arabidopsis* [6], *Populus* [20], *Punica granatum* [35], and *Glycine max* [36], highlighting considerable variability in TALE gene numbers across different species [20,35]. For instance, there are 103 *TALE* genes in *Switchgrass*, 33 in *Arabidopsis*, 27 in *Oryza sativa*, and only 18 in *Ananas comosus*. *TALE* genes regulate diverse physiological processes, including fruit development in *Solanum lycopersicum* [37] and stress responses in *Cucumis sativus* [38] and *Triticum aestivum* [24]. In this study, our genome-wide analysis identified 23 *PeTALE* genes—comparable to *Prunus armeniaca* (22 genes) but slightly higher than *Vitis vinifera* (21 genes)—with predicted nuclear localization (Table 1) and conserved chromosomal distribution patterns (Figure S1). The identified *PeTALE* genes are conserved in other *Passiflora* species with varying floral morphologies. Based on the well-established roles of KNOX and BELL genes in meristem identity, floral organogenesis, and hormone responses, it is likely that functional conservation exists across the *Passiflora* genus, due to the high sequence similarity observed. This point has been incorporated into phylogenomic studies across the *Passiflora* genus to better understand the link between gene diversification and floral morphology. Phylogenetic classification resolved these genes into nine clades (four KNOX and five BELL), mirroring evolutionary patterns observed in dicots, where KNAT subgroups are retained but lost in monocots (Figure 1). Structural conservation of the homeodomain motifs (Homeobox-KN, KNOX1/2, and ELK) further supports their functional roles in DNA-binding and transcriptional regulation [6]. Specifically, the C-terminal homeobox-KN domain facilitates DNA interaction, while the ELK domain directs nuclear localization [12,22,39,40]. These findings corroborate the functional conservation of TALE proteins across angiosperms, while also highlighting lineage-specific adaptations in *P. edulis*.

### 3.2. PeTALEs in Floral Organogenesis: From Meristem Dynamics to Organ-Specific Regulation

Synteny analysis identified 18 collinear *PeTALE* pairs, predominantly within the BELL subfamily, with tandem duplication events contributing significantly to *TALE* gene expansion. The expansion of *BELL* genes may be associated with the evolutionary adaptation of *Passiflora* to its unique reproductive strategy and complex floral structures. Comparative genomics suggest that lineage-specific duplications of *BELL* genes could underlie the developmental specialization necessary for the elaborate coronas, ovules, and stigmas observed in *Passiflora*. This dominance likely reflects an evolutionary bias favoring BELL-mediated transcriptional regulation during floral organ identity specification. The expansion of BELL genes appears tightly linked to the increased floral complexity of *Passiflora*. BELL proteins are known to form heterodimers with KNOX proteins to regulate developmental boundaries and meristem maintenance. In *Passiflora*, this interaction may have been co-opted to refine the differentiation of additional floral whorls, thereby contributing to the species’ intricate floral structures. This suggests a functional co-evolution between PeBELL genes and floral architectural innovations.

Purifying selection (*Ka*/*Ks* < 1) likely preserved the core developmental functions of these genes during evolution. Inter-species comparisons revealed stronger syntenic conservation with *Arabidopsis* than with monocots, supporting conserved regulatory roles in dicot development. Gene structure analysis demonstrated conserved intron–exon arrangements within the PeTALE family, consistent with findings in other studies such as *Populus* [20]. Moreover, genomic architecture analysis showed that all identified *PeTALE* genes possess at least one intron, with highly conserved structural features across family members. Protein motif analysis confirmed that members within the same subfamily share identical motifs, reinforcing the notion of functional conservation [41]. Promoter profiling uncovered abundant *cis*-elements linked to light responsiveness (45.83%), phytohormone signaling (18.75%), and stress adaptation (18.75%), including low-temperature-responsive elements (LTRs) critical for cold stress regulation. This aligns with *P. edulis*’s sensitivity to temperature fluctuations [20,34], suggesting that *PeTALEs* may orchestrate transcriptional responses to temperature fluctuations via LTR-mediated pathways.

### 3.3. Temperature-Responsive PeTALEs: Balancing Development and Stress Adaptation

Expression profiling across floral developmental stages revealed that *PeTALE* genes exhibit clear functional specialization. During early organogenesis, *PeTALE8*/*9* showed peak expression during the initiation of petals, coronas, stigmas, and ovules, paralleling the roles reported for *BEL1* homologs in ovule development [40,42]. Tissue-specific regulation was evident, with *PeTALE21* displaying strong expression during the early stages of corona development, suggesting a critical role in corona formation. Similarly, *PeTALE23*/*16*/*4*/*22* showed preferential expression in sepals, echoing findings from *Populus* where TALE factors are involved in organ patterning [20]. In terms of maturation control, *PeTALE13*/*14*/*16* exhibited progressive upregulation during petal and corona development, suggesting their involvement in the maturation of these floral organs. Recent studies have further highlighted the roles of *TALE* genes in reproductive development: in pomegranate, *TALE* genes were predicted to regulate SAM, flower, and ovule development [35], while in walnut, *TALE* gene expression varied across flower bud developmental stages [43]. In cotton, the downregulation of the *TALE* family member *GhSTM3* affected flowering time [44]. While *KNOX1* genes such as *STM* and *KNAT1* in *Arabidopsis* are largely restricted to meristematic tissues, certain *PeTALE* genes (*PeBELL7* and *PeKNOX3*) exhibit expression in floral organs such as the ovary and corona, suggesting a divergence in spatial regulation. This may reflect neofunctionalization events post-duplication that allowed *PeTALE* genes to acquire novel roles in reproductive development.

Temperature stress significantly modulated the expression of several *PeTALE* genes. For instance, the genes *PeTALE3*/*4* (BELL subfamily) were upregulated under cold stress, while *PeTALE10*/*18* (KNOX subfamily) responded preferentially to heat stress. These findings highlight the potential roles of the *PeTALE* genes in mediating plant response to environmental stress. The cold-induced expression of *PeTALE3/4* correlates with the presence of LTR cis-elements in their promoters, suggesting a mechanism for transcriptional reprogramming under chilling stress—a key adaptive trait for *P. edulis*, which is highly sensitive to bud abortion under cold temperatures [33,34]. Conversely, the heat-activated expression of *PeTALE10/18* may help counteract thermal damage, potentially through mechanisms similar to wheat *TaKNOX11a*’s role in mitigating oxidative stress [45]. This functional dichotomy mirrors findings in *Gossypium* and *Prunus*, where *TALE* genes differentially regulate responses to abiotic stresses such as drought and salt stress [37,45,46], further supporting their pleiotropic roles in stress adaptation. We hypothesize that *BELL* genes like *PeTALE3* and *PeTALE4* mediate cold stress response in *P. edulis*, enhancing flower bud resilience under low temperatures. Moreover, temperature-sensitive PeTALEs may act as molecular hubs, integrating environmental signals with hormonal pathways. For example, *PeBELL2* and *PeKNOX5*, which are downregulated under elevated temperatures, may modulate GA biosynthesis repressors or interact with ABA-responsive promoter elements, thereby influencing flowering time and organ differentiation under fluctuating thermal conditions.

In conclusion, this study provides a comprehensive analysis of the *PeTALE* gene family in *Passiflora edulis*, shedding light on their roles in floral organ development and stress adaptation. However, further functional studies are necessary to fully elucidate the regulatory networks and molecular mechanisms underlying these processes. Such investigations will be crucial for understanding how *P. edulis* adapts to environmental challenges and how *TALE* genes orchestrate complex developmental programs, ultimately informing future crop improvement strategies.

## 4. Materials and Methods

### 4.1. Identification of Putative TALE Gene Members in P. edulis

The *P. edulis* genome and proteome datasets were obtained from the National Genomics Data Center (NGDC, accession number GWHAZTM00000000, https://ngdc.cncb.ac.cn/ (accessed on 25 September 2024)) [47]. To identify putative *TALE* gene members within the *P. edulis* genome, we employed two complementary approaches: Hidden Markov Model (HMM) searching and BLAST-based sequence alignment. For the HMM approach, the TALE family domain (PF00046) from the Pfam database (www.pfam.org (accessed on 25 September 2024)) was used to scan the *P. edulis* protein sequences using HMMER3 software (v3.4, http://hmmer.janelia.org/ (accessed on 25 September 2024)) [48]. Simultaneously, a BLASTP v2.12.0 search was conducted using known plant TALE protein sequences retrieved from the NCBI database as query sequences. Sequences with an E-value threshold of <1 × 10^−5^ were retained for further analysis. Non-redundant candidates from both methods were consolidated and subsequently subjected to domain validation using SMART (http://smart.embl-heidelberg.de (accessed on 27 September 2024)) [49] and the Conserved Domain Database (CDD, www.ncbi.nlm.nih.gov/cdd (accessed on 28 September 2024)) [50]. Only sequences containing both a homeodomain (HD) and either KNOX1/KNOX2 (for the KNOX subfamily) or a BELL/POX (for the BELL subfamily) gene were retained. Based on domain composition, the identified genes were classified into two major subfamilies: the KNOX subfamily, characterized by the presence of HD and KNOX1/KNOX2 domains, and the BELL subfamily, characterized by HD and BELL/POX domains. In total, 23 *TALE* genes were identified and named according to their chromosomal positions in the *P. edulis* genome for consistency. To predict their potential cellular roles, subcellular localization was assessed using WoLF PSORT (https://wolfpsort.hgc.jp (accessed on 16 November 2024)). Additionally, the key physicochemical properties of the encoded proteins, including the molecular weight (MW), isoelectric point (pI), and grand average of hydropathicity (GRAVY), were calculated using the ExPASy v3.0 online tools [51].

### 4.2. Sequence Alignment and Phylogenetic Analyses

To elucidate the evolutionary relationships of *TALE* genes, a comparative phylogenetic analysis was conducted across multiple plant species. Full-length coding sequences of *P. edulis TALE* genes were extracted using TBtools-II (v 2.119) [52]. TALE protein sequences from other plant species, including *Oryza sativa*, *Vitis vinifera*, and *Arabidopsis thaliana*, were retrieved from the PlantTFDB v4.0 database (http://planttfdb.gao-lab.org/ (accessed on 16 December 2024)). For sequence alignment, multiple sequence alignments of the TALE proteins were performed using the MUSCLE algorithm implemented in MEGA 11 [53], with manual curation to optimize the representation of conserved domains. Based on the aligned sequences, a phylogenetic tree was constructed using the neighbor-joining (NJ) method in MEGA 11, with bootstrap support values calculated from 1000 replicates to assess the reliability of the tree topology. The resulting phylogenetic tree was subsequently annotated and visualized using the iTOL web tool (https://itol.embl.de (accessed on 30 January 2025)) [54].

### 4.3. Chromosomal Localization and Duplication Analysis of TALE Genes

The chromosomal locations of *TALE* genes in *P. edulis* were identified using the passion fruit genome annotation file, and the genes were subsequently mapped to their respective chromosomes. Visualization of chromosomal localization was performed using TBtools software (v 2.119) [52], providing a clear representation of the distribution of *TALE* genes across the genome. To investigate the evolutionary dynamics of the *P. edulis* genome, particularly regarding gene duplication events, a gene duplication analysis was conducted using the Multiple Collinearity Scanning Toolkit (MCScanX) [55] with default settings. Genomic data for *Oryza sativa*, *Arabidopsis thaliana*, and *Vitis vinifera* were retrieved from the JGI Phytozome database (https://phytozome-next.jgi.doe.gov (accessed on 15 February 2025)) for comparative analysis. A comparative synteny approach was employed to assess orthologous relationships and gene duplication events between *TALE* genes in *P. edulis* and these three reference species. Syntenic blocks of TALE superfamily members across species were visualized using TBtools, highlighting conserved chromosomal regions and identifying potential evolutionary conserved gene clusters.

To further understand the selective pressures acting on *TALE* genes, *Ka* (non-synonymous) and *Ks* (synonymous) substitution rates were calculated for tandem and segmental duplication events using TBtools [52]. Selection modes were inferred based on *Ka*/*Ks* ratios: *Ka*/*Ks* > 1 indicated positive selection, *Ka*/*Ks* < 1 suggested purifying selection, and *Ka*/*Ks* = 1 reflected neutral evolution. Furthermore, the divergence times of segmental duplications were estimated using the formula T = *Ks*/(2λ), assuming a synonymous substitution rate (λ) of 6.5 × 10^−9^ substitutions per site per year [52].

### 4.4. Conserved Motif Prediction and Cis-Acting Element Analysis

Conserved protein motifs within the TALE members were identified using the MEME suite (https://meme-suite.org/meme/ (accessed on 23 February 2025)) [56], with the following parameters: maximum number of motifs set to 10, and motif width ranging from 6 to 50 residues. Exon–intron structures of the TALE genes were determined by aligning their coding sequences to the corresponding genomic DNA sequences using the genome annotation GFF3 files. The conserved regions within the TALE domains were further verified through the CDD database (www.ncbi.nlm.nih.gov/cdd (accessed on 23 February 2025)) [50].

To explore potential regulatory elements governing the expression of *TALE* genes, 2000 bp genomic sequences upstream of each gene’s translation start site were extracted as putative promoter regions. Cis-regulatory elements within these regions were predicted using PlantCARE (http://bioinformatics.psb.ugent.be/webtools/plantcare/html (accessed on 27 February 2025)) [57], followed by manual curation to annotate elements associated with stress responses, hormonal signaling, and developmental regulation.

### 4.5. Tertiary Structure Analysis of TALE Family Member

To investigate the three-dimensional (tertiary) structures of TALE proteins, all protein sequences were submitted to the SWISS-MODEL server (https://swissmodel.expasy.org (accessed on 8 March 2025)) [58] using a homology-based modeling approach, with template selection guided by the Protein Data Bank (PDB, www.rcsb.org (accessed on 12 March 2025)). Secondary structural elements, including alpha-helices, beta-sheets, and loops, were annotated based on alignments with the selected reference models. After the structural models were generated, the resulting three-dimensional configurations were refined and visualized using PyMOL v3.1 [59] to analyze domain architecture, protein fold patterns, and potential functional motifs.

### 4.6. Plant Material and Sample Preparation

Healthy two-month-old *P. edulis* (cultivar Qinmi) plants were cultivated in controlled-climate chambers at the Institute of Horticulture, Guangxi Academy of Agricultural Sciences, China. Temperature stress treatments were conducted following the protocol outlined by An et al. (2024) [47]. For cold stress, plants were transferred to a growth chamber set to 20 °C, while for heat stress, they were exposed to 30 °C. Floral buds were sampled at various time intervals to capture the stress response: 12, 24, and 48 h for cold and heat stresses. Control plants were maintained at 25 °C throughout the experiment. Three biological replicates were performed. Floral buds were collected at the designated time points, immediately frozen in liquid nitrogen, and stored at −80 °C. Total RNA was extracted from 100 mg samples using the RNAprep Pure Plant Kit (Tiangen Biotech (Beijing) Co., Ltd., Beijing, China) according to the manufacturer’s protocol. RNA quality and concentration were assessed using NanoDrop 2000 spectrophotometry (Thermo Fisher, Waltham, MA, USA) (A260/A280 > 1.8) and confirmed by agarose gel electrophoresis.

### 4.7. RNA Isolation, qRT-PCR, and Expression Pattern Analysis

Total RNA was isolated from *P. edulis* tissues using the RNAprep Pure Plant Kit (Tiangen Biotech) according to the manufacturer’s instructions. RNA concentrations were determined, and 1 µg of RNA per biological replicate was used for PCR library construction. Sequencing libraries were prepared using the NEBNext Ultra RNA Library Prep Kit (NEB, Beverly City, MA, USA) for Illumina platforms. RNA integrity was verified using a NanoDrop 2000 spectrophotometer (Thermo Fisher Scientific) and confirmed by agarose gel electrophoresis. The expression levels of *PeTALE* genes were assessed by synthesizing cDNA from total RNA using the ThermoScript RT-PCR kit (Thermo Fisher Scientific, Carlsbad, CA, USA). qRT-PCR was conducted to analyze gene expression across various floral developmental stages, including Bracts1−8 (br1−br8), Sepals 1−8 (Se1−Se8), Petals 1−8 (Pe1−Pe8), Corona1−8 (Ca1p−Ca8), Stamens1,8,9 (St1, St8 and St9), Stigma1−8 (Sg1−Sg8), and Ovules 2−8 (Ov2−Ov8). All samples were collected and immediately frozen in liquid nitrogen for subsequent RNA extraction. Bar graphs were generated using GraphPad Prism 10.1.2 software. Relative gene expression was calculated using the 2^−∆∆Ct^ method, with statistical significance indicated by * (*p* ≤ 0.05) and ** (*p* ≤ 0.01), as determined by Student’s *t*-test.

Four *PeTALE* genes (*PeTALE15*, *16*, *19*, and *22*) from the KNOX-II, BELL-II, BELL-I, and BELL-IV clusters, respectively, were selected for qRT-PCR validation based on their notable expression profiles identified in the RNA-seq analysis. These genes exhibited distinct expression patterns in reproductive organs or under temperature treatments, suggesting potential involvement in floral morphogenesis and stress-responsive regulatory pathways. For qRT-PCR, reactions were performed under the following conditions: initial denaturation at 95 °C for 30 s, followed by 40 cycles of 95 °C for 10 s and 60 °C for 30 s. Each 20 µL reaction contained 1 µL of cDNA template, 10 µL of 2 × Taq Pro Universal SYBR qPCR Master Mix (Vazyme, Nanjing, China), 0.4 µL of each primer (10 µM), and 8.2 µL of ddH_2_O. Amplifications were conducted using a Bio-Rad Real-time PCR system (Foster City, CA, USA), with three biological replicates per sample. *EF1α* was used as the reference gene for normalization [47].

For RNA-seq analysis, raw reads were pre-processed to obtain clean reads using Trimmomatic software v3.06 with default settings. Cleaned reads were subsequently mapped to the *P. edulis* reference genome using Hisat2 software v2.2.1. Gene expression levels were quantified by calculating FPKM (fragments per kilobase of transcript per million mapped reads) values using Cufflinks. Log2-transformed FPKM values were used to determine the expression patterns of *PeTALE* genes, and a heatmap was generated using TBtools software [52].

### 4.8. Protein–Protein Interaction (PPI) Network Construction of PeTALEs

To predict potential protein–protein interactions (PPIs) among *P. edulis* TALE proteins, we utilized the Search Tool for the Retrieval of Interacting Genes/Proteins (STRING) database (http://cn.string-db.org/ (accessed on 15 March 2025)), a well-established platform for exploring molecular interactions. Candidate PPI pairs were identified using the STRING database, with a combined confidence score greater than 0.4, reflecting a moderate level of interaction confidence. The resulting PPI data were visualized and analyzed by constructing an interaction network using Cytoscape (v3.8.2, https://cytoscape.org/ (accessed on 15 March 2025)), a powerful tool for network analysis and visualization. This approach enabled us to map the interaction relationships among PeTALE proteins and infer their potential functional roles in cellular processes based on network connectivity patterns.

### 4.9. Yeast Two-Hybrid Assay

To validate the predicted interactions, we employed the yeast two-hybrid (Y2H) system to detect direct protein–protein interactions in vivo. The full-length CDSs of *PeTALE2*, *PeTALE5*, and *PeTALE7* were individually cloned into the pGADT7 activation domain vector at the *Nde*I restriction site. Similarly, the full-length CDSs of *PeTALE4*, *PeTALE32*, and *PeTALE6* were cloned into the pGBKT7 DNA-binding domain vector at the *Nde*I site. The bait–prey pairs were co-transformed into the yeast strain AH109 using the Matchmaker™ GAL4 Two-Hybrid System (Takara Bio, USA, Inc., San Jose, CA, USA), alongside controls: pGADT7-T + pGBKT7-53 as the positive control and pGADT7-T + pGBKT7-lam as the negative control. After transformation, yeast cells were incubated at 28 °C for 3 days. To select for successful transformations, colonies were first plated onto selective medium (SD/-Leu-Trp) to ensure plasmid integration. Protein–protein interactions were then assessed by plating the transformants onto a more stringent selection medium (SD/-Leu-Trp-His-Ade) supplemented with 3-amino-1,2,4-triazole (3-AT) to inhibit non-specific background activation.

### 4.10. Statistical Analysis

The experimental data were analyzed using a one-way analysis of variance (ANOVA) with SPSS 24.0. All results are presented as the mean ± standard error. Significant differences (*p* < 0.05) between the control and treatment groups were further assessed using Student’s *t*-test.

## 5. Conclusions

This study provides a comprehensive analysis of the *PeTALE* gene family in *Passiflora edulis*, identifying 23 distinct *PeTALE* genes. Phylogenetic and syntenic analyses revealed that both tandem and segmental duplications contributed to the expansion and evolutionary diversification of the *PeTALE* gene family in passion fruit. Tissue- and stage-specific expression patterns suggest that *PeTALE* genes are crucial regulators of reproductive development, particularly in floral organ formation and in response to temperature stresses. Moreover, our findings highlight the critical role of *PeTALE* genes in coordinating floral organ development and modulating floral bud differentiation under varying temperature conditions. This work establishes a solid foundation for future research aimed at elucidating the molecular mechanisms by which *PeTALE* genes influence flower and fruit development, as well as their potential roles in stress adaptation.

## Figures and Tables

**Table 1:** The *PeTALE* gene members and their physicochemical property characteristics of *P. edulis*.

Gene Name	Gene ID	Chromosome	Size (aa)	MW (kDa)	pI	Instability Index	A.I.	GRAVY	Predicted Location
*PeTALE1*	P_edulia010000512.g	LG01	393	44.913	5.45	39.81	68.78	−0.524	Nucleus
*PeTALE2*	P_edulia010000849.g	LG01	632	68.668	6.49	42.76	67.45	−0.609	Nucleus
*PeTALE3*	P_edulia010001174.g	LG01	307	34.041	6.84	41.00	69.90	−0.579	Nucleus
*PeTALE4*	P_edulia010001451.g	LG01	610	67.089	8.70	42.84	63.33	−0.649	Nucleus
*PeTALE5*	P_edulia010003102.g	LG01	315	35.834	4.70	45.00	74.73	−0.723	Nucleus
*PeTALE6*	P_edulia010004158.g	LG01	414	47.280	6.04	53.44	60.12	−0.858	Nucleus
*PeTALE7*	P_edulia010004162.g	LG01	378	43.216	6.21	52.59	60.95	−0.865	Nucleus
*PeTALE8*	P_edulia010004665.g	LG01	463	51.731	5.85	45.85	82.59	−0.419	Nucleus
*PeTALE9*	P_edulia010004708.g	LG01	463	51.796	5.90	44.65	79.85	−0.455	Nucleus
*PeTALE10*	P_edulia010004901.g	LG01	347	38.850	6.02	44.83	66.40	−0.505	Nucleus
*PeTALE11*	P_edulia020006626.g	LG02	195	22.506	6.66	56.48	64.00	−0.923	Nucleus
*PeTALE12*	P_edulia020006883.g	LG02	731	80.057	7.02	48.53	71.70	−0.616	Nucleus
*PeTALE13*	P_edulia020007397.g	LG02	660	73.090	5.50	51.09	65.94	−0.622	Nucleus
*PeTALE14*	P_edulia020007434.g	LG02	672	74.178	5.59	52.11	65.06	−0.618	Nucleus
*PeTALE15*	P_edulia050012005.g	LG05	396	44.497	7.35	55.42	83.99	−0.484	Nucleus
*PeTALE16*	P_edulia060015581.g	LG06	747	82.694	6.03	46.17	70.12	−0.622	Nucleus
*PeTALE17*	P_edulia060015582.g	LG06	466	52.262	6.28	53.85	81.65	−0.449	Nucleus
*PeTALE18*	P_edulia070016949.g	LG07	304	34.545	4.95	45.36	60.33	−0.718	Nucleus
*PeTALE19*	P_edulia070018156.g	LG07	599	65.746	5.76	45.63	78.85	−0.385	Nucleus
*PeTALE20*	P_edulia070018309.g	LG07	500	55.357	6.18	43.87	73.40	−0.549	Nucleus
*PeTALE21*	P_edulia080019381.g	LG08	811	89.581	6.16	47.89	67.36	−0.618	Nucleus
*PeTALE22*	P_edulia080019551.g	LG08	689	75.049	8.75	47.31	69.38	−0.603	Nucleus
*PeTALE23*	P_edulia080019849.g	LG08	675	74.569	5.68	47.60	68.24	−0.631	Nucleus

**Table 2:** The *Ka*/*Ks* ratios of the duplicated *PeTALE* gene pairs.

Duplicated Gene Pairs	*Ka*	*Ks*	*Ka*/*Ks*	Group	Duplicated Type
*PeTALE1*/*PeTALE5*	0.31	2.29	0.14	KNOX-III/KNOX-III	Segmental
*PeTALE2*/*PeTALE4*	0.13	0.65	0.21	BELL-IV/BELL-IV	Segmental
*PeTALE8*/*PeTALE9*	0.01	0.02	0.48	BELL-V/BELL-V	tandem
*PeTALE8*/*PeTALE17*	0.13	0.53	0.25	BELL-V/BELL-V	Segmental
*PeTALE13*/*PeTALE14*	0.01	0.02	0.39	BELL-V/BELL-V	tandem
*PeTALE13*/*PeTALE16*	0.51	2.81	0.18	BELL-V/BELL-V	Segmental
*PeTALE13*/*PeTALE23*	0.12	0.54	0.23	BELL-V/BELL-V	Segmental
*PeTALE14*/*PeTALE23*	0.12	0.51	0.24	BELL-V/BELL-V	Segmental
*PeTALE12*/*PeTALE22*	0.13	0.6	0.21	BELL-IV/BELL-IV	Segmental
*PeTALE16*/*PeTALE23*	0.48	1.92	0.25	BELL-V/BELL-V	Segmental

## Data Availability

Data are contained within the article.

## Supplementary Materials

The following supporting information can be downloaded at https://www.mdpi.com/article/10.3390/plants14101440/s1: Figure S1: Chromosome locations of *PeTALE*s; Table S1: The TALE-related locus in synteny analysis within passion fruit genome; Table S2: The TALE-related locus in synteny analysis among *Arabidopsis*, passion fruit, grape, and rice.
