# Supplementary material for: Functional Characterization of KNOX and BELL Genes in Temperature-Responsive Floral Morphogenesis of Passion Fruit (Passiflora edulis)
Source: Plants (Basel). 2025 May 12;14(10):1440. doi: 10.3390/plants14101440 (PMC12114980; doi:10.3390/plants14101440)
Supplement: Supplementary file 1 [file plants-14-01440-s001.zip › plants-3602604-supplementary.pdf]

Functional Characterization of KNOX and BELL Genes in Temperature-Responsive Floral Morphogenesis of Passion Fruit (*Passiflora edulis*)

Xinni Jiang [1], Jie Miao [1], Weifan Zu [1], Ruohan Zhou [1], Lexin Zheng [1], Ying Wei [1], Chunmei Lai [1], Rongjuan Qin [2], Ping Zheng [1,3], Xiuqing Wei [4], Jiahui Xu [4], Yuan Qin [1,3,*] and Xiaoping Niu [1,*]

[1] Fujian Provincial Key Laboratory of Haixia Applied Plant Systems Biology, College of Life Science, Fujian Agriculture and Forestry University, Fuzhou 350002, China; 17850636650@163.com (X.J.); 15280688801@163.com (J.M.); z739136369@163.com (W.Z.); 15306758678@163.com (R.Z.); lulu1424142044@163.com (L.Z.); weiying0702@163.com (Y.W.); cmlai21@fafu.edu.cn (C.L.); zhengping13@mails.ucas.ac.cn (P.Z.)

[2] Fishery Multiplication Management Station of Lijiang River Water Supply Hub Project, Guilin 541001, China; qinrj6898@163.com

[3] Pingtan Science and Technology Research Institute, Fujian Agriculture and Forestry University, Pingtan 350400, China

[4] Fruit Research Institute, Fujian Academy of Agricultural Sciences, Fuzhou 350013, China; weixiuqing47@foxmail.com (X.W.); xjhui577@163.com (J.X.)

* Correspondence: yuanqin@fafu.edu.cn (Y.Q.); xpniu0613@126.com (X.N.)

Supplemental Figure S1. Chromosome locations of *PeTALE*s. The 23 *PeTALE* genes in *Passiflora edulis* are distributed across 9 pairs of chromosomes, with only 6 chromosomes containing these gene. Notably, the distribution is non-random, as certain chromosomes exhibit dense clusters of *PeTALE* genes in specific regions. This pattern implies that gene duplication events have contributed to the expansion of the *PeTALE* gene family within the *P. edulis* genome.

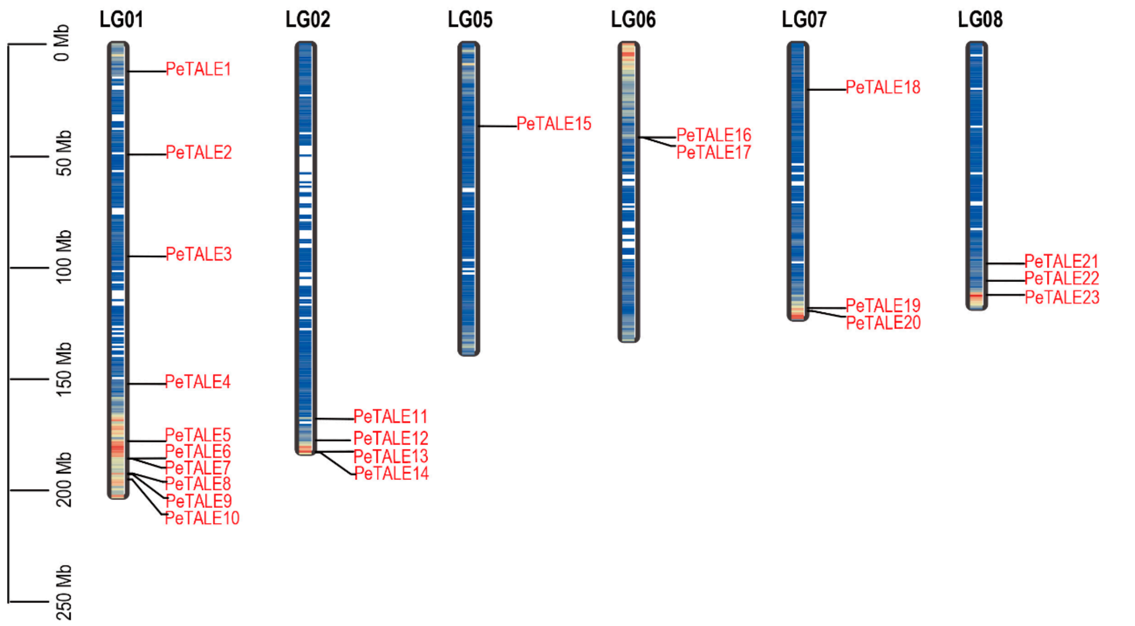

Supplemental Tables

Table S1. The TALE-related locus in synteny analysis within passion fruit genome.

Gene ID	Chrom	Name
P_edulia010004665.g/P_edulia010004708.g	LG01/LG01	PeTALE8/PeTALE9
P_edulia010000849.g/P_edulia010001451.g	LG01/LG01	PeTALE2/PeTALE4
P_edulia010000512.g/P_edulia010003102.g	LG01/LG01	PeTALE1/PeTALE5
P_edulia010004665.g/P_edulia020007397.g	LG01/LG02	PeTALE8/PeTALE13
P_edulia010004708.g/P_edulia020007434.g	LG01/LG02	PeTALE9/PeTALE13
P_edulia010004708.g/P_edulia020007397.g	LG01/LG02	PeTALE9/PeTALE14
P_edulia010004665.g/P_edulia020007434.g	LG01/LG02	PeTALE8/PeTALE14
P_edulia010004665.g/P_edulia060015582.g	LG01/LG06	PeTALE8/PeTALE17
P_edulia010004708.g/P_edulia060015581.g	LG01/LG06	PeTALE9/PeTALE16
P_edulia010004665.g/P_edulia080019849.g	LG01/LG08	PeTALE8/PeTALE23
P_edulia010004708.g/P_edulia080019849.g	LG01/LG08	PeTALE9/PeTALE23
P_edulia010000849.g/P_edulia080019551.g	LG01/LG08	PeTALE2/PeTALE22
P_edulia020007397.g/P_edulia020007434.g	LG02/LG02	PeTALE13/PeTALE14
P_edulia020007397.g/P_edulia060015581.g	LG02/LG06	PeTALE13/PeTALE16
P_edulia020007397.g/P_edulia080019849.g	LG02/LG08	PeTALE13/PeTALE23
P_edulia020007434.g/P_edulia080019849.g	LG02/LG08	PeTALE14/PeTALE23
P_edulia020006883.g/P_edulia080019551.g	LG02/LG08	PeTALE12/PeTALE22
P_edulia060015581.g/P_edulia080019849.g	LG06/LG08	PeTALE16/PeTALE23

Table S2. The TALE-related locus in synteny analysis among *Arabidopsis*, passion fruit, grape and rice

Gene ID	Chromosome	Name
P_edulia010004665.g/transcript:AT1G75430.1	Pe-01/At-1	PeTALE8/AtTALE11
P_edulia010003102.g/transcript:AT1G70510.2	Pe-01/At-1	PeTALE5/AtTALE8
P_edulia010000512.g/transcript:AT1G23380.2	Pe-01/At-1	PeTALE1/AtTALE5
P_edulia010003102.g/transcript:AT1G23380.2	Pe-01/At-1	PeTALE5/AtTALE5
P_edulia010004901.g/transcript:AT1G62360.1	Pe-01/At-1	PeTALE10/AtTALE6
P_edulia010000512.g/transcript:AT1G70510.2	Pe-01/At-1	PeTALE1/AtTALE8
P_edulia010004708.g/transcript:AT1G19700.3	Pe-01/At-1	PeTALE9/AtTALE3
P_edulia010000849.g/transcript:AT2G35940.2	Pe-01/At-2	PeTALE2/AtTALE20
P_edulia010001451.g/transcript:AT5G02030.1	Pe-01/At-2	PeTALE4/AtTALE20
P_edulia010001174.g/transcript:AT5G02030.1	Pe-01/At-5	PeTALE3/AtTALE29
P_edulia020007434.g/transcript:AT1G75410.2	Pe-02/At-1	PeTALE14/AtTALE7
P_edulia020007434.g/transcript:AT1G19700.3	Pe-02/At-1	PeTALE14/AtTALE10
P_edulia020007397.g/transcript:AT1G75410.2	Pe-02/At-1	PeTALE13/AtTALE3
P_edulia020007397.g/transcript:AT2G16400.1	Pe-02/At-1	PeTALE13/AtTALE10
P_edulia020007434.g/transcript:AT2G16400.1	Pe-02/At-2	PeTALE14/AtTALE3
P_edulia020007397.g/transcript:AT2G16400.1	Pe-02/At-2	PeTALE13/AtTALE12
P_edulia020007434.g/transcript:AT4G34610.2	Pe-02/At-4	PeTALE14/AtTALE12
P_edulia020007397.g/transcript:AT4G34610.2	Pe-02/At-4	PeTALE13/AtTALE26
P_edulia050012005.g/transcript:AT1G62990.1	Pe-05/At-1	PeTALE15/AtTALE26
P_edulia060015581.g/transcript:AT1G75410.2	Pe-06/At-1	PeTALE16/AtTALE10
P_edulia060015581.g/transcript:AT1G19700.3	Pe-06/At-1	PeTALE16/AtTALE3
P_edulia060015581.g/transcript:AT4G34610.2	Pe-06/At-4	PeTALE16/AtTALE12
P_edulia070018309.g/transcript:AT4G32040.1	Pe-07/At-4	PeTALE20/AtTALE17
P_edulia070018156.g/transcript:AT4G32980.1	Pe-07/At-4	PeTALE19/AtTALE26
P_edulia070018309.g/transcript:AT5G11060.1	Pe-07/At-5	PeTALE20/AtTALE23
P_edulia070018309.g/transcript:AT5G25220.1	Pe-07/At-5	PeTALE20/AtTALE24
P_edulia080019849.g/transcript:AT1G75410.2	Pe-08/At-1	PeTALE23/AtTALE30
P_edulia080019849.g/transcript:AT1G19700.3	Pe-08/At-1	PeTALE23/AtTALE31
P_edulia080019849.g/transcript:AT2G16400.1	Pe-08/At-2	PeTALE23/AtTALE10
P_edulia080019551.g/transcript:AT2G27220.2	Pe-08/At-2	PeTALE22/AtTALE3
P_edulia080019849.g/transcript:AT4G34610.2	Pe-08/At-4	PeTALE23/AtTALE26
P_edulia010003102.g/GSVIVT01013790001	Pe-01/Vv-1	PeTALE5/VvTALE9
P_edulia010004901.g/GSVIVT01030488001	Pe-01/Vv-12	PeTALE10/VvTALE16
P_edulia010001174.g/GSVIVT01016458001	Pe-01/Vv-13	PeTALE3/VvTALE10
P_edulia010003102.g/GSVIVT01007715001	Pe-01/Vv-17	PeTALE5/VvTALE2
P_edulia010000512.g/GSVIVT01007715001	Pe-01/Vv-17	PeTALE1/VvTALE2
P_edulia010004665.g/GSVIVT01009779001	Pe-01/Vv-18	PeTALE8/VvTALE5
P_edulia010004708.g/GSVIVT01009779001	Pe-01/Vv-18	PeTALE9/VvTALE5
P_edulia010004158.g/GSVIVT01009273001	Pe-01/Vv-18	PeTALE6/VvTALE3
P_edulia010001174.g/GSVIVT01034073001	Pe-01/Vv-8	PeTALE3/VvTALE18
P_edulia010000849.g/GSVIVT01011146001	Pe-01/Vv-8	PeTALE2/VvTALE7

P_edulia050012005.g/GSVIVT01019880001	Pe-05/Vv-2	PeTALE15/VvTALE13
P_edulia020006626.g/GSVIVT01012897001	Pe-02/Vv-11	PeTALE11/VvTALE8
P_edulia020007397.g/GSVIVT01009781001	Pe-02/Vv-18	PeTALE13/VvTALE6
P_edulia020007434.g/GSVIVT01009781001	Pe-02/Vv-18	PeTALE14/VvTALE6
P_edulia020007434.g/GSVIVT01024224001	Pe-02/Vv-3	PeTALE14/VvTALE14
P_edulia020007397.g/GSVIVT01024224001	Pe-02/Vv-3	PeTALE13/VvTALE14
P_edulia020006626.g/GSVIVT01035921001	Pe-02/Vv-4	PeTALE11/VvTALE20
P_edulia020006883.g/GSVIVT01037575001	Pe-02/Vv-6	PeTALE12/VvTALE21
P_edulia080019849.g/GSVIVT01009781001	Pe-08/Vv-18	PeTALE23/VvTALE6
P_edulia080019849.g/GSVIVT01024224001	Pe-08/Vv-3	PeTALE23/VvTALE14
P_edulia080019381.g/GSVIVT01025220001	Pe-08/Vv-6	PeTALE21/VvTALE15
P_edulia080019551.g/GSVIVT01037575001	Pe-08/Vv-6	PeTALE22/VvTALE21
P_edulia070016949.g/GSVIVT01031241001	Pe-07/Vv-14	PeTALE18/VvTALE17
P_edulia070018156.g/GSVIVT01035361001	Pe-07/Vv-4	PeTALE19/VvTALE19
P_edulia070018309.g/GSVIVT01035921001	Pe-07/Vv-4	PeTALE20/VvTALE20
P_edulia060015582.g/GSVIVT01009779001	Pe-06/Vv-18	PeTALE17/VvTALE5
P_edulia060015581.g/GSVIVT01009781001	Pe-06/Vv-18	PeTALE16/VvTALE6
P_edulia010001174.g/Os01g62920.1	Pe-01/Os-4	PeTALE3/OsTALE2
P_edulia010001451.g/Os11g06020.1	Pe-01/Os-11	PeTALE4/OsTALE41
P_edulia010004158.g/Os03g51690.2	Pe-01/Os-3	PeTALE6/OsTALE19
P_edulia010004158.g/Os03g47016.1	Pe-01/Os-3	PeTALE6/OsTALE12
P_edulia010001174.g/Os05g38120.1	Pe-01/Os-5	PeTALE3/OsTALE31
P_edulia020006626.g/Os02g08544.1	Pe-02/Os-2	PeTALE11/OsTALE3
P_edulia020006626.g/Os06g43860.1	Pe-02/Os-6	PeTALE11/OsTALE34
P_edulia020006626.g/Os08g19650.1	Pe-02/Os-8	PeTALE11/OsTALE38
P_edulia070018309.g/Os02g08544.1	Pe-07/Os-2	PeTALE20/OsTALE3
P_edulia070018156.g/Os02g13310.1	Pe-07/Os-2	PeTALE19/OsTALE6
P_edulia070018309.g/Os06g43860.1	Pe-07/Os-6	PeTALE20/OsTALE34
